# Supplementary figures and images for: Genetic and chemical validation of Plasmodium falciparum aminopeptidase PfA-M17 as a drug target in the hemoglobin digestion pathway
Source: eLife. 2022 Sep 13;11:e80813. doi: 10.7554/eLife.80813 (PMC9470162; doi:10.7554/eLife.80813)

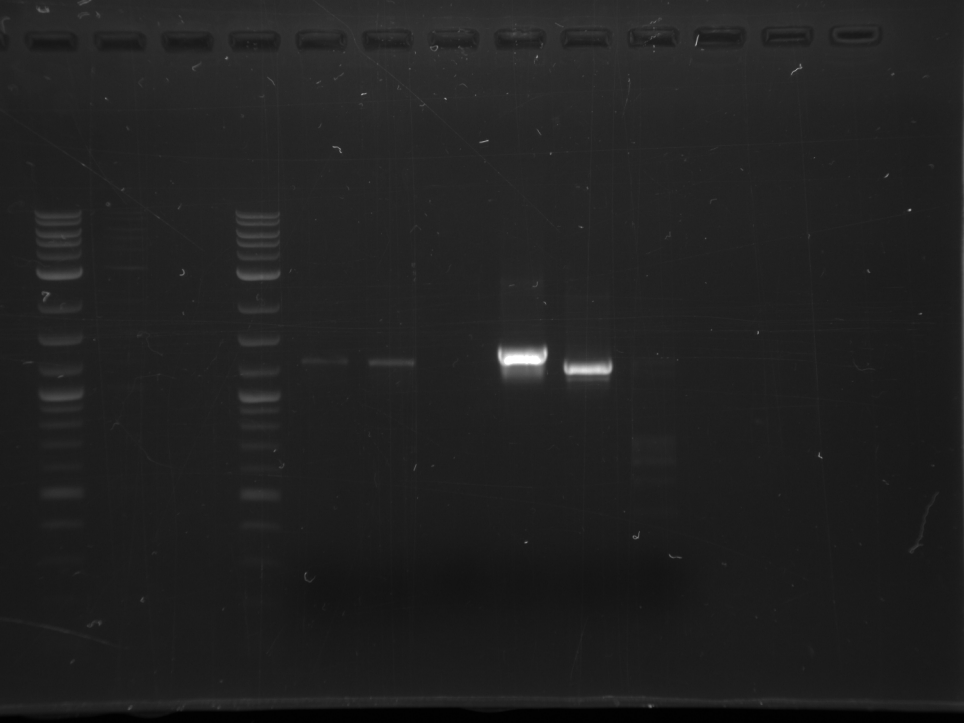

Supplement: Figure 1—source data 1. [file elife-80813-fig1-data1.pdf]

Kb

2 -

1.5 -

1 -

3D7 WT

M17-HAgImS

3D7 WT

M17-HAgImS

DO733 +  
DO276

DO733 +  
DO734

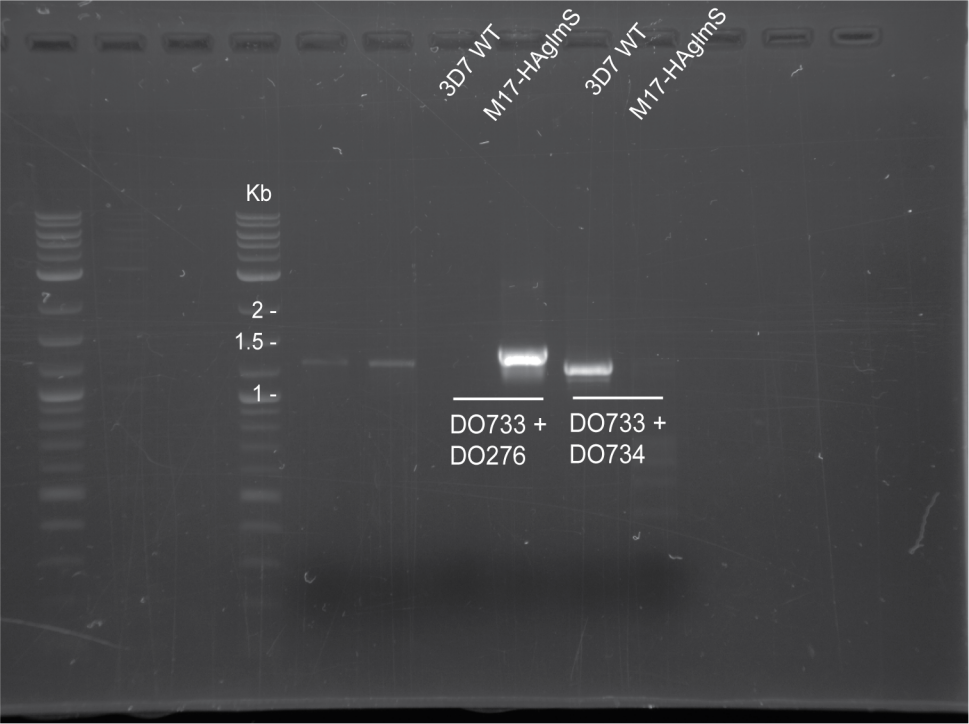

Supplement: Figure 1—source data 2. [file elife-80813-fig1-data2.pdf]

Kb

3D7 WT  
M17-HAglms

1.5 -  
1.2 -  
1 -

DO657 +  
DO658

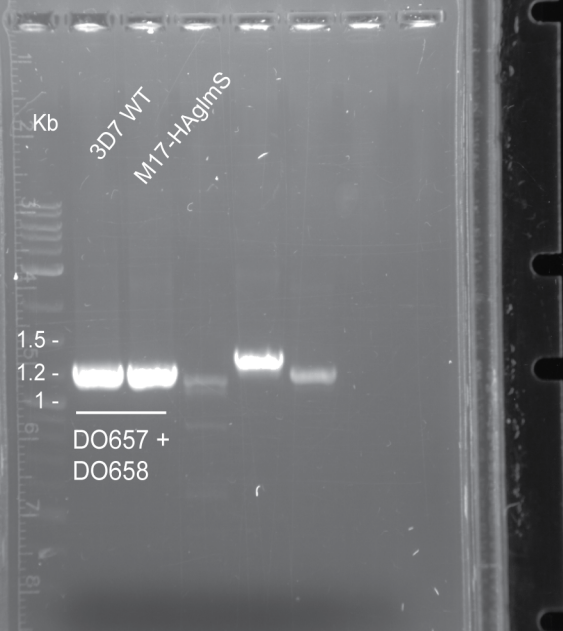

Supplement: Figure 1—source data 4. [file elife-80813-fig1-data4.pdf]

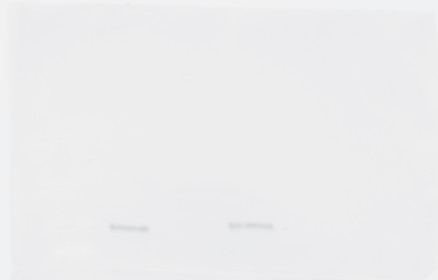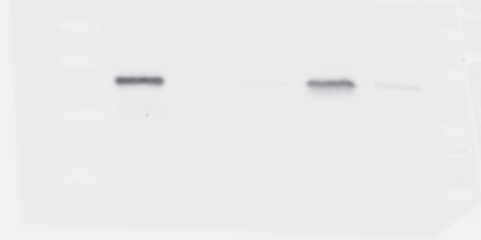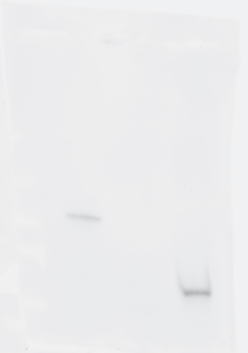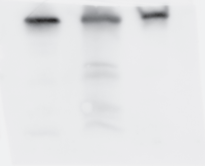

Supplement: Figure 1—source data 5. [file elife-80813-fig1-data5.pdf]

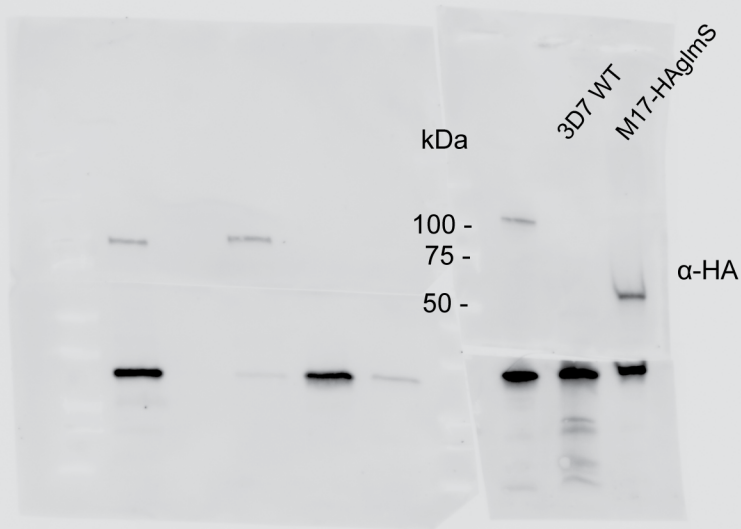

Supplement: Figure 1—source data 6. [file elife-80813-fig1-data6.pdf]

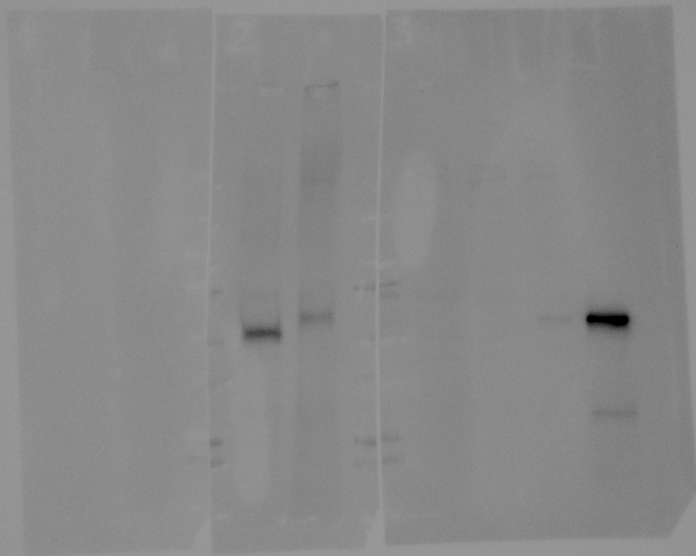

Supplement: Figure 1—figure supplement 1—source data 1. [file elife-80813-fig1-figsupp1-data1.pdf]

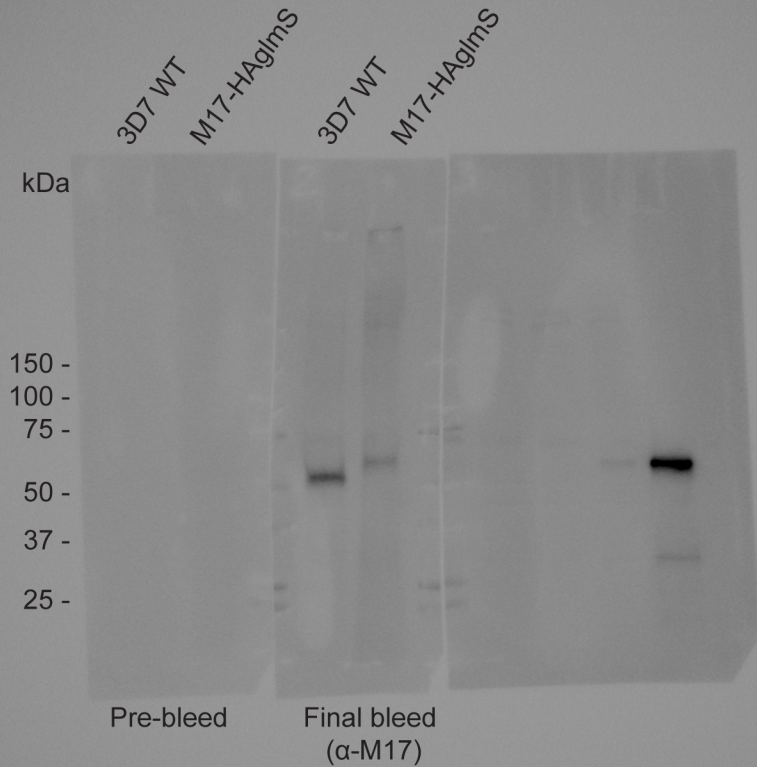

Supplement: Figure 1—figure supplement 1—source data 2. [file elife-80813-fig1-figsupp1-data2.pdf]

Starting material  
Hypotonic  
 $\text{Na}_2\text{CO}_3$   
TX-100  
Insoluble

kDa

100 -  
75 -

$\alpha$ -HSP101

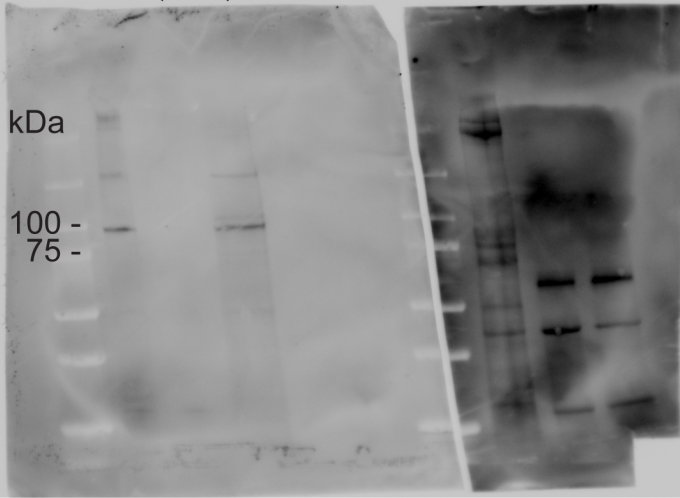

Supplement: Figure 2—source data 2. [file elife-80813-fig2-data2.pdf]

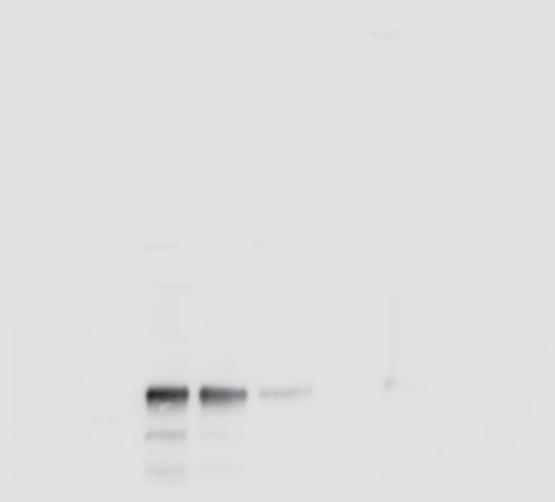

Supplement: Figure 2—source data 3. [file elife-80813-fig2-data3.pdf]

kDa

Saponin

Hypotonic

Na<sup>2</sup>CO<sub>3</sub>

TX-100

Insoluble

37 -

25 -

α-GAPDH

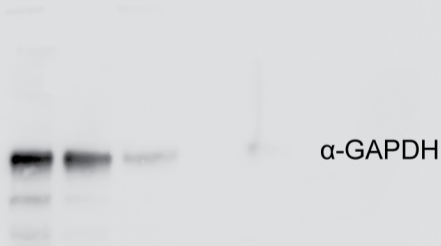

Supplement: Figure 2—source data 4. [file elife-80813-fig2-data4.pdf]

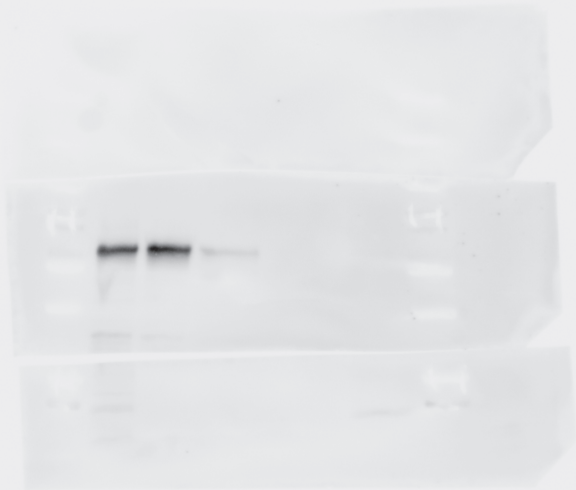

Supplement: Figure 2—source data 5. [file elife-80813-fig2-data5.pdf]

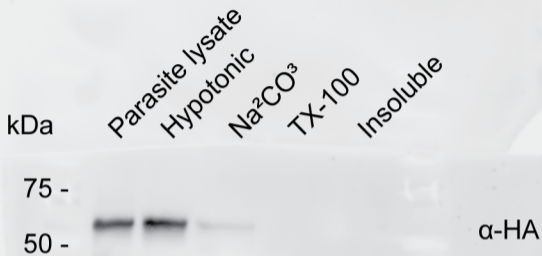

Supplement: Figure 2—source data 6. [file elife-80813-fig2-data6.pdf]

kDa    Parasite lysate  
         Hypotonic  
          $\text{Na}_2\text{CO}_3$   
         TX-100  
         Insoluble

37 -

25 -

$\alpha$ -EXP2

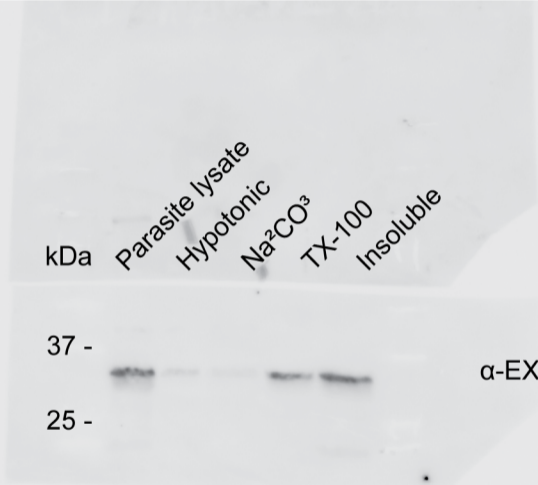

Supplement: Figure 2—source data 8. [file elife-80813-fig2-data8.pdf]

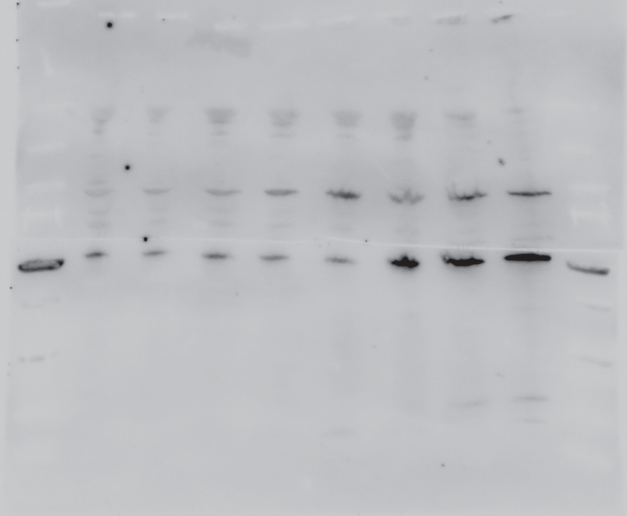

Supplement: Figure 2—source data 9. [file elife-80813-fig2-data9.pdf]

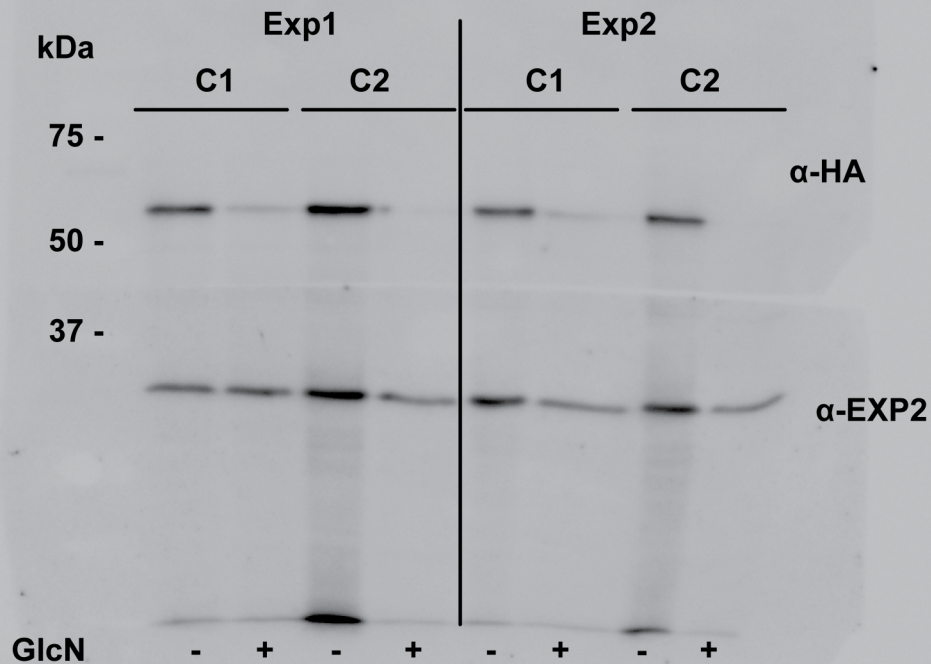

Supplement: Figure 3—source data 2. [file elife-80813-fig3-data2.pdf]
