# Supplementary material for: Genetic and chemical validation of Plasmodium falciparum aminopeptidase PfA-M17 as a drug target in the hemoglobin digestion pathway
Source: eLife. 2022 Sep 13;11:e80813. doi: 10.7554/eLife.80813 (PMC9470162; doi:10.7554/eLife.80813)
Supplement: Figure 2—source data 10. [file elife-80813-fig2-data10.pdf]

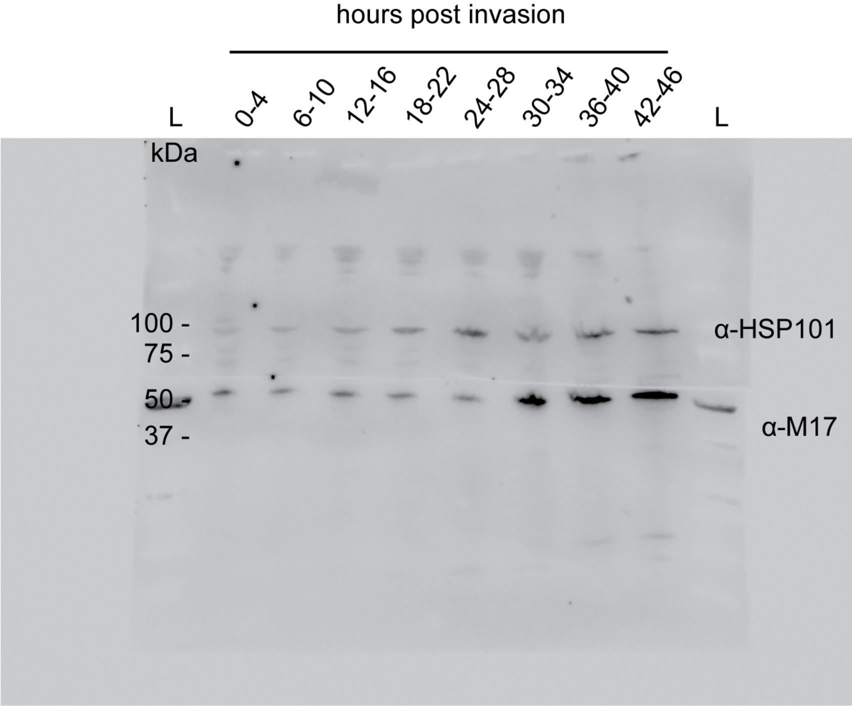
hours post invasion
L
0-4
6-10
12-16
18-22
24-28
30-34
36-40
42-46
L
kDa
100 -
75 -
50 -
37 -
α-HSP101
α-M17
