# Supplementary material for: Genetic and chemical validation of Plasmodium falciparum aminopeptidase PfA-M17 as a drug target in the hemoglobin digestion pathway
Source: eLife. 2022 Sep 13;11:e80813. doi: 10.7554/eLife.80813 (PMC9470162; doi:10.7554/eLife.80813)
Supplement: Supplementary file 1. [file elife-80813-supp1.docx]

**Supplementary File 1. Crystallography and refinement statistics for *Pf*A-M17 bound to 3**

|  | *Pf*A-M17-**3** |
| --- | --- |
| PDB ID | 7RIE |
| *Data Collection Statistics* |  |
| Wavelength | 0.953727 |
| Resolution range | 48.73 - 2.49 (2.58 - 2.49) |
| Space group | P 21 21 21 |
| Unit cell (a, b, c, α, β, γ) | 175.29 175.86 234.68 90 90 90 |
| Total reflections | 498247 (47143) |
| Unique reflections | 250277 (23847) |
| Multiplicity | 2.0 (2.0) |
| Completeness (%) | 99.45 (95.51) |
| Mean I/sigma(I) | 7.02 (1.30) |
| Wilson B-factor | 41.46 |
| R-pim | 0.06067 (0.3973) |
| CC1/2 | 0.996 (0.727) |
| *Refinement statistics* |  |
| Reflections used in refinement | 250101 (23835) |
| Reflections used for R-free | 12429 (1229) |
| R-work | 0.1947 (0.2850) |
| R-free | 0.2281 (0.3315) |
| Number of non-hydrogen atoms | 0.964 (0.818) |
| macromolecules | 0.952 (0.737) |
| ligands | 49071 |
| solvent | 47016 |
| Protein residues | 528 |
| RMS(bonds) | 1527 |
| RMS(angles) | 6182 |
| Ramachandran favored (%) | 0.003 |
| Ramachandran allowed (%) | 0.57 |
| Ramachandran outliers (%) | 97.07 |
| Rotamer outliers (%) | 2.78 |
| Clashscore | 0.15 |
| Average B-factor | 1.17 |
| macromolecules | 2.99 |
| ligands | 43.18 |
| solvent | 43.34 |
| Number of TLS groups | 41.48 |

Statistics for the highest-resolution shell are shown in parentheses.
