# Supplementary material for: Genetic and chemical validation of Plasmodium falciparum aminopeptidase PfA-M17 as a drug target in the hemoglobin digestion pathway
Source: eLife. 2022 Sep 13;11:e80813. doi: 10.7554/eLife.80813 (PMC9470162; doi:10.7554/eLife.80813)
Supplement: Supplementary file 2. [file elife-80813-supp2.docx]

**Supplementary File 2. Oligonucleotide sequences used in this study**

| Name | Target | Oligonucleotide Sequence |
| --- | --- | --- |
| DO276 | Ribozyme | GTGATTTCTCTTTGTTCAAGGA |
| DO657 | M17 | AATAACCGCGGGGATCAGTTGCTGATTTAAGT |
| DO658 | M17 | AATAAGGCGCGCCCTAGAGCGTCATTGAGTACAA |
| DO733 | M17 | GACCGATGAAAGGTTCAAATCT |
| DO734 | M17 | GTGAGGTACACCACACATG |
